# Identification microenvironment immune features and key genes in elderly stroke patients

**DOI:** 10.1097/MD.0000000000033108

**Published:** 2023-03-03

**Authors:** Yisheng Peng, Zhengli Liu, Guanqi Fu, Boxiang Zhao, Maofeng Gong, Zhaoxuan Lu, Yangyi Zhou, Liang Chen, Haobo Su, Wensheng Lou, Guoping Chen, Xu He, Jianping Gu, Jie Kong

**Affiliations:** a Department of Radiological Intervention, Women’s Hospital of Nanjing Medical University, Nanjing Maternity and Child Health Care Hospital, Nanjing, Jiangsu, P.R. China; b Department of Interventional Radiology, Nanjing First Hospital, Nanjing Medical University, Nanjing, Jiangsu, P.R. China.

**Keywords:** gene ontology, hub gene, immune infiltration, protein interaction maps, stroke

## Abstract

The purpose of this study was to identify the signaling pathways and immune microenvironments related to elderly stroke patients.

**Methods:**

We downloaded the public transcriptome data (GSE37587) from the gene expression omnibus and divided the patients into young and old groups and identified differentially expressed genes (DEGs). Gene ontology function analysis, Kyoto encyclopedia of genes and genomes pathway analysis, and gene set enrichment analysis (GSEA) were performed. A protein-protein interaction network was constructed and hub genes were identified. Gene-miRNA, gene-TF, and gene-drug networks were constructed using the network analyst database. The immune infiltration score was evaluated using single-sample gene set enrichment analysis GSEA, its correlation with age was computed and visualized using R software.

**Results:**

We identified 240 DEGs, including 222 upregulated and 18 downregulated DEGs. Gene ontology enrichment was significantly enriched in response to the virus, type I interferon signaling pathway, cytological component, focal adhesion, cell-substrate adherents junction, and the cytosolic ribosome. GSEA identified the following mechanisms: heme metabolism, interferon gamma response, and interferon alpha response. Ten hub genes included interferon alpha-inducible protein 27, human leucocyte antigen-G, interferon-induced protein with tetratricopeptide repeats 2, 2’-5’-oligoadenylate synthetase 2, interferon alpha-inducible protein 6, interferon alpha-inducible protein 44-like, interferon-induced protein with tetratricopeptide repeats 3, interferon regulatory factor 5, myxovirus resistant 1, and interferon-induced protein with tetratricopeptide repeats 1. Quantitative analysis of immune infiltration showed that increased age was significantly positively correlated with myeloid-derived suppressor cells and natural killer T cells, and negatively correlated with immature dendritic cells.

**Conclusion:**

The present research could help us better understand the molecular mechanisms and immune microenvironment of elderly patients with stroke.

## 1. Introduction

Population aging in the Western world is gradually accelerating.^[1]^ Ischemic heart disease and stroke are the main causes of death in the United States, their incidence rate increases exponentially with age.^[1]^ Epidemiological studies have shown that aging is an independent risk factor for cardiovascular diseases. There are 4 independent risk factors for stroke in patients, including prior stroke or transient ischemic attacks, advancing age, hypertension, and diabetes.^[2]^ The research from S. S. Ganguly and his colleague showed that the leading independent risk factors for ischemic Stroke include: Family history of stroke, hypertension, and reduced high-density lipoprotein.^[3]^ However, the mechanism underlying the effect of aging on stroke has not been fully elucidated.

Datasets GSE37587 were downloaded to identify differentially expressed genes (DEGs) between young and old stroke patients. Next, the R software was used to perform enrichment analysis. Furthermore, protein-protein interactions (PPI), hub genes, gene-miRNA networks, gene-TF networks, gene-drug networks, and immune infiltration were analyzed to explore the mechanisms associated with aging stroke patients.

## 2. Materials and Methods

### 2.1. Microarray data

The gene expression profile of GSE37587 was downloaded from the database (gene expression omnibus; http://www.ncbi.nlm.nih.gov/geo/). This dataset was based on the GPL6883 platform (Illumina HumanRef-8 v3.0 expression beadchip). The GSE37587 dataset contained 68 samples, including 46 patients who were ≥ 65 years old, and were defined as the old group, and 22 of them were younger than 65 years old and were defined as the young group.

The study was approved by the Ethics Committee of the Nanjing First Hospital.

### 2.2. Intragroup data repeatability test

Pearson relationship test was used to evaluate repeatability. All measurable processing and graphics were performed using R programming. The correlations between all samples were visualized using the heatmap package in R. Principal component analysis (PCA) was performed to evaluate sample relationships and variability.

### 2.3. Identification of DEGs

DEGs between the young and old groups were identified using the Bioconductor R package limma. The cutoff value was set at *P* < .05, and |logFC| > 0.5. A total of 240 DEGs were analyzed from 18631 genes, including 222 upregulated and 18 downregulated genes. A heatmap of DEGs was created using R based on the package “ComplexHeatmap.”

### 2.4. Functional enrichment of DEGs by gene ontology (GO), Kyoto encyclopedia of genes and genomes (KEGG), and gene set enrichment analysis (GSEA) analysis

The cluster Profiler package was used to perform GO, KEGG, and GSEA analyses and visualize the results of the enrichment analyses. GO enrichment analysis was performed for the ontology categories. KEGG pathway analysis, a commonly used bioinformatics database (https://www.kegg.jp/), was performed using the cluster Profiler package.^[4]^ GSEA was used to identify the statistically enriched genes and was also measured using the cluster profiler package.

### 2.5. Construction and analysis of the PPI network and identification of hub genes

The search tool for the retrieval of interacting gene (STRING) database (https://string-db.org/) was searched to predict the PPI based on the imported DEGs.^[5]^ Then, the free programming Cytoscape (http://cytoscape.org/) was used to visualize the PPI organization. Hub genes were identified based on the DNMC score calculated using the CytoHubba plug-in.^[6]^

### 2.6. Hub gene-miRNA network and the hub gene-TF network construction

To identify putative miRNA and transcription factors regulating hub genes, we used network analyst (https://www.networkanalyst.ca/) to integrate miRNA databases miRTar base (http://mirtarbase.mbc.nctu.edu.tw/) and TF databases ENCODE (http://cistrome.org/BETA/).^[7]^ Gene, miRNA, and gene-TF networks were constructed and visualized using these databases.

### 2.7. DEGs-drug network construction

To identify potential drugs, we applied network analyst (https://www.networkanalyst.ca/) and the Drug Bank database (Version 5.0) (https://go.drugbank.com/) to construct the DEGs-drug network. All those drug-gene interaction networks were visualized by network analyst.^[7]^

### 2.8. Analysis of immune infiltration

Quantitative analysis of immune infiltration was performed with the GSVA package using single-sample gene set enrichment analysis (ssGSEA) to estimate the abundance of different types of immune cells. The Spearman correlation between age and the different types of immune cell ssGSEA scores were calculated and visualized using R software with the packages ggplot2, ggrepel, and ggstatsplot.

## 3. Results

### 3.1. Validation of the dataset

**Figure 1.**
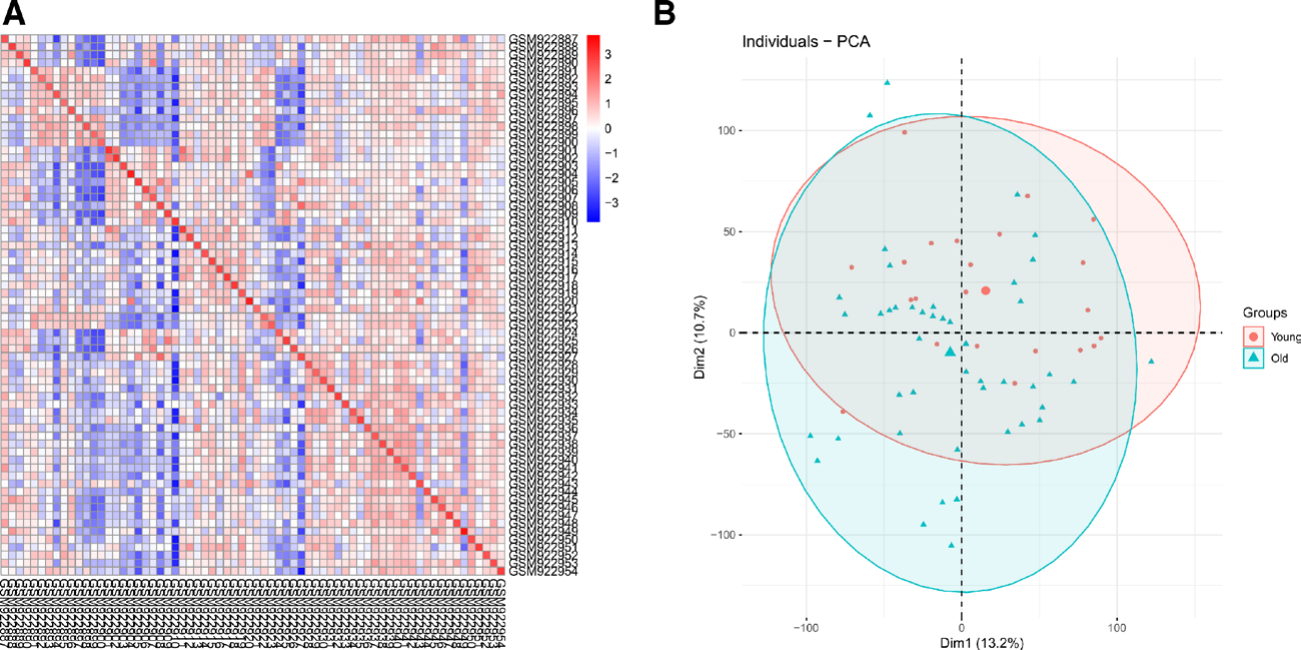
The intrasample data repeatability of GSE37587 was tested by Pearson correlation analysis and PCA. (A) Pearson correlation analysis between all samples from the GSE37587 dataset was analyzed. The correlation coefficient was reflected by varying color intensities. (B) The PCA was performed to analyze all samples from the GSE37587 dataset. The X-axis represents the PC1 and the y-axis represents the PC2. PCA = principal component analysis, PC1 = principal component 1, PC2 = principal component 2.

We used both Pearson correlation test and PCA to test the relationship between the young and old groups. The heatmap of the GSE37587 dataset indicated that there were strong correlations between the old and young groups (Fig. 1A). The PCA analyses of GSE37587 are displayed in Figure 1B, which shows the distances between the old and young groups in both PC1 and PC2.

### 3.2. Identification of DEGs

**Figure 2.**
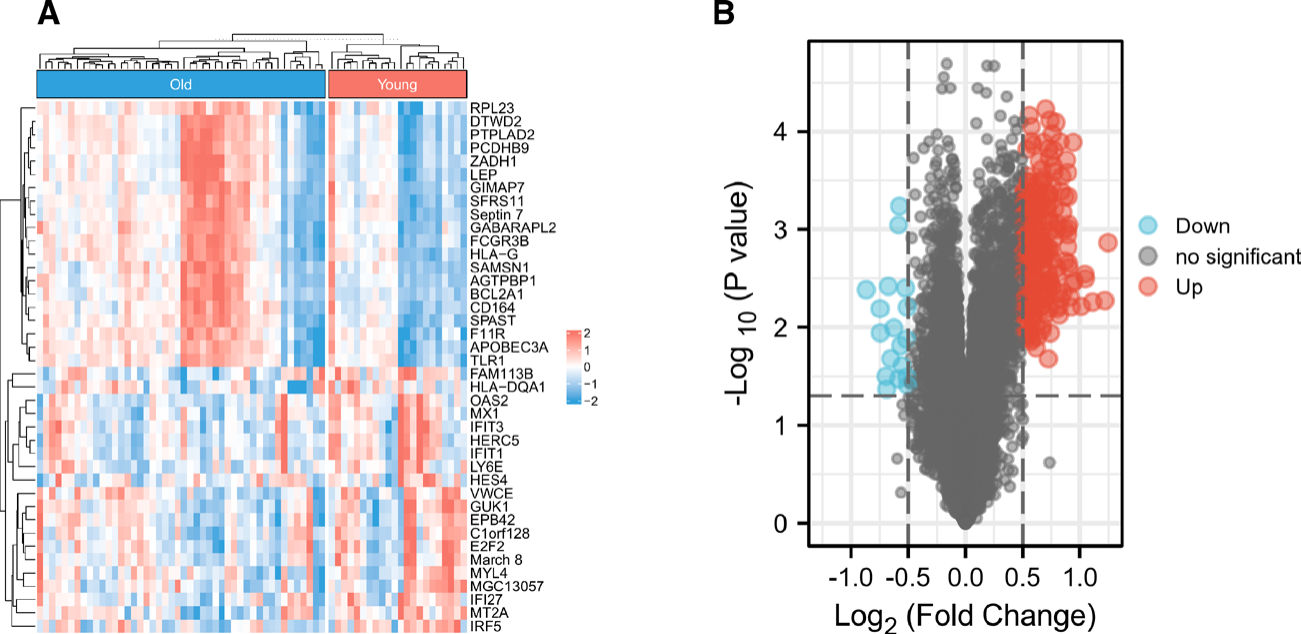
Identification of DEGs between Old samples and young samples. (A) The significant DEGs between the 2 groups are represented by a heatmap. (B) A volcano plot of DEGs between 2 groups were created after analysis of the GSE37587 dataset. DEGs = differentially expressed genes.

An aggregate of 240 DEGs was found to be dependent on *P* < .05 and |logFC| > 0.5, with 222 upregulated and 18 downregulated genes. Both the heatmap (Fig. 2A) and volcano plots (Fig. 2B) were used to show the DEGs.

### 3.3. Functional and pathway enrichment analysis

**Figure 3.**
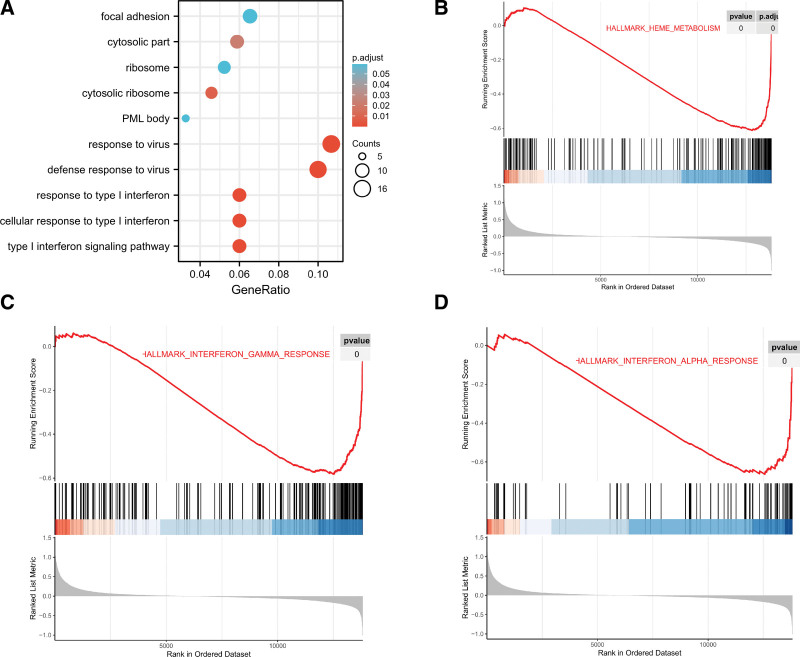
GO enrichment analysis of DEGs and Gene set enrichment analysis (GSEA) analysis of the GSE37587 dataset. (A) The biological process (BP) and cellular component (CC) analysis of GO enrichment analysis. (B–D) The top 3 pathways enriched by the GSEA, the heme metabolism, interferon gamma response, and interferon alpha response pathways were enriched. DEGs = differentially expressed genes. GO = gene ontology, GSEA = gene set enrichment analysis.

GO was enriched in response to the type I interferon signaling pathway. Regarding cytological component, focal adhesion, cell-substrate adherents junction, and cytosolic ribosome were enriched (Fig. 3A). No KEGG terms were enriched by DEGs. The GSEA analysis identified possible mechanisms including heme metabolism, interferon gamma response, and interferon alpha response (Fig. 3B–D).

### 3.4. Construction of the PPI network and identification of hub genes

**Figure 4.**
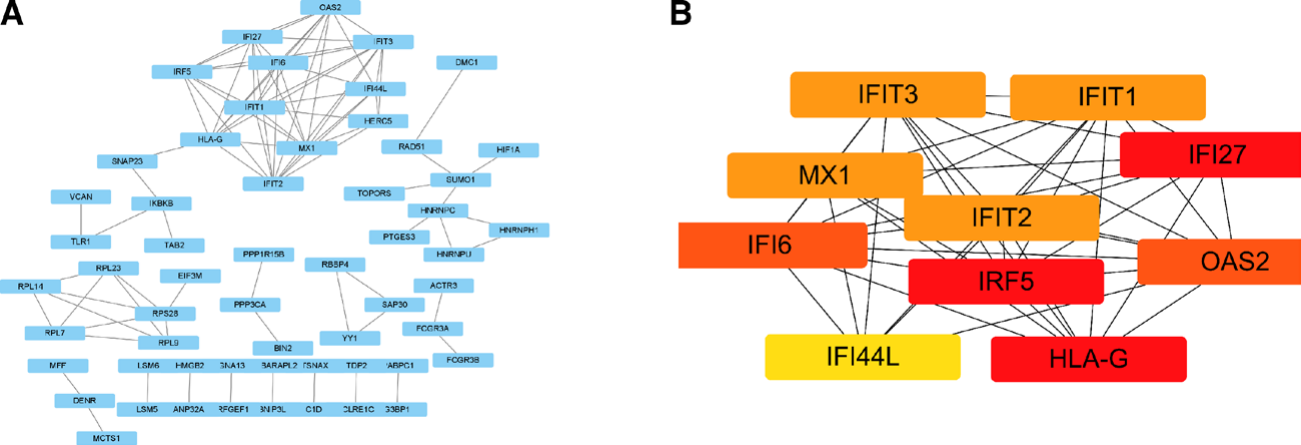
PPI network and hub genes network. (A) STRING database was used to construct the PPI network. (B) The hub genes included IFI27, HLA-G, IFIT2, OAS2, IFI6, IFI44L, IFIT3, IRF5, MX1, and IFIT1. HLA-G = human leucocyte antigen-G, IFI27 = interferon alpha-inducible protein 27, IFI44L = interferon alpha-inducible protein 44-like, IF16 = interferon alpha-inducible protein 6, IFIT1 = interferon-induced protein with tetratricopeptide repeats 1, IFIT2 = interferon-induced protein with tetratricopeptide repeats 2, IFIT3 = interferon-induced protein with tetratricopeptide repeats 3, IRF5 = interferon regulatory factor 5, MX1 = myxovirus resistant 1, OAS2 = 2’-5’-oligoadenylate synthetase 2, PPI = protein-protein interaction, STRING = search tool for the retrieval of interacting gene.

As shown in Figure 4A, the PPI network of DEGs was constructed by the STRING database (https://string-db.org/, STRING) and visualized through the software Cyto scape.^[6]^ The top 10 hub genes are interferon alpha-inducible protein 27 (IFI27), human leucocyte antigen-G, interferon-induced protein with tetratricopeptide repeats 2 (IFIT2), 2’-5’-oligoadenylate synthetase 2 (OAS2), interferon alpha-inducible protein 6 (IFI6), interferon alpha-inducible protein 44-like, interferon-induced protein with tetratricopeptide repeats 3 (IFIT3), interferon regulatory factor 5 (IRF5), myxo virus resistant 1, and interferon-induced protein with tetratricopeptide repeats 1 (Fig. 4B).

### 3.5. Construction of the target gene-miRNA network and the target gene-TF network

**Figure 5.**
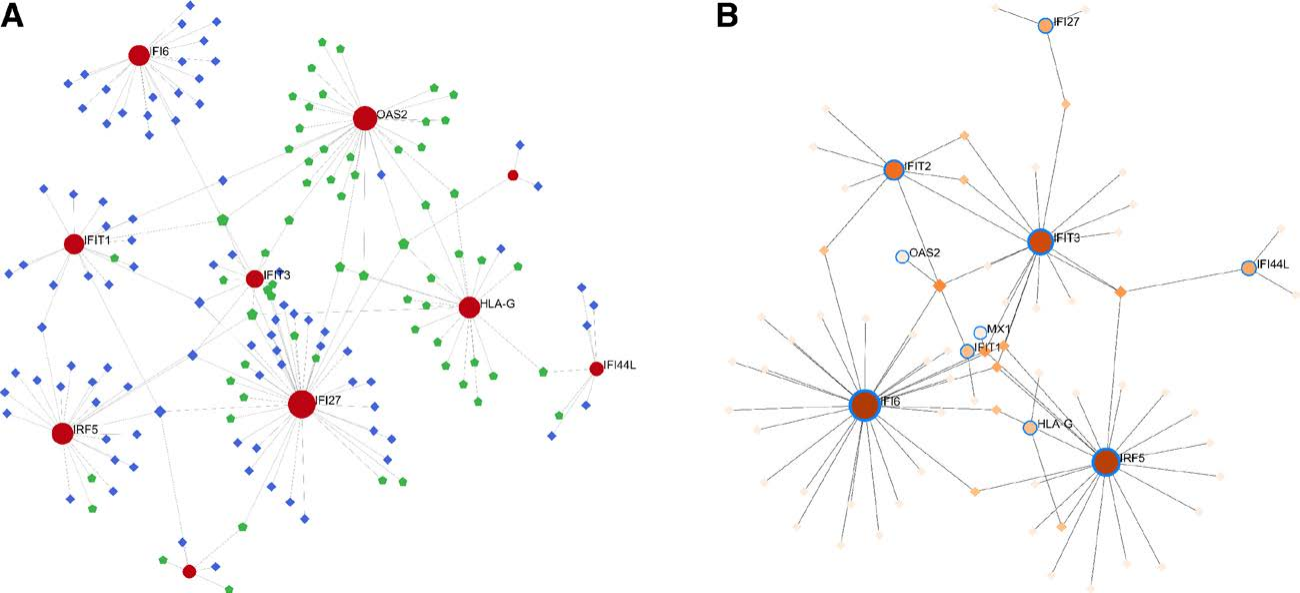
The target gene-miRNA networks (A) and the target gene-TF networks (B).

The top 3 DEGs modulated by miRNAs were IFI27 regulated by 43 miRNAs, OAS2 regulated by 30 miRNAs, and IRF5 regulated by 22 miRNAs. The miRNA that regulated the largest number of DEGs (4 genes) was hsa-mir-146a-5p (Fig. 5A). The top 5 targeted DEGs modulated by TFs were IFI6 (28 TFs), IRF5 (20 TFs), IFIT3 (16 TFs), IFIT2 (8 TFs), and IRF1(5 TFs) (Fig. 5B).

### 3.6. Identification of the potential drugs

**Figure 6.**
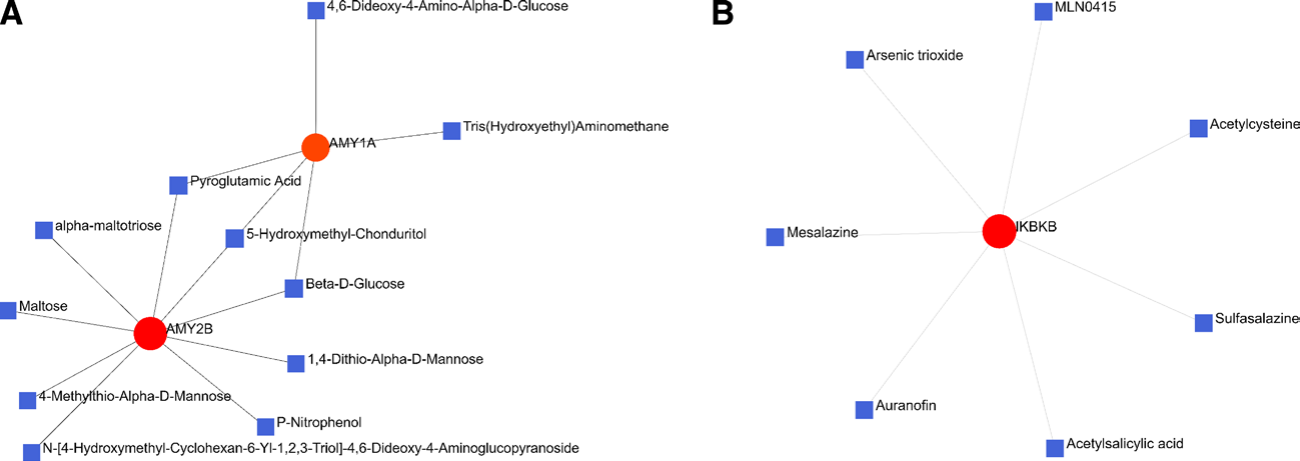
The target gene-drug networks. The red circle indicates the gene and the blue diamond node indicates the drug.

The Drug Bank database was used to identify potential drugs or molecular compounds targeted at elderly stroke patients. As shown in the drug-gene interaction network (Fig. 6), 11 drugs or molecular compounds, including beta-d-glucose, pyroglutamic acid, and 5-hydroxymethyl-chondrite, could regulate the expression of AMY2B and AMY1A. In addition, 7 drugs or molecular compounds, such as mesalazine, sulfasalazine, and acetylsalicylic acid, could interact with IKBKB. Furthermore, 4 drugs or molecular compounds, including octreotide, vapreotide, and pasireotide, regulated SSTR2.

### 3.7. Evaluation of the immune infiltration

**Figure 7.**
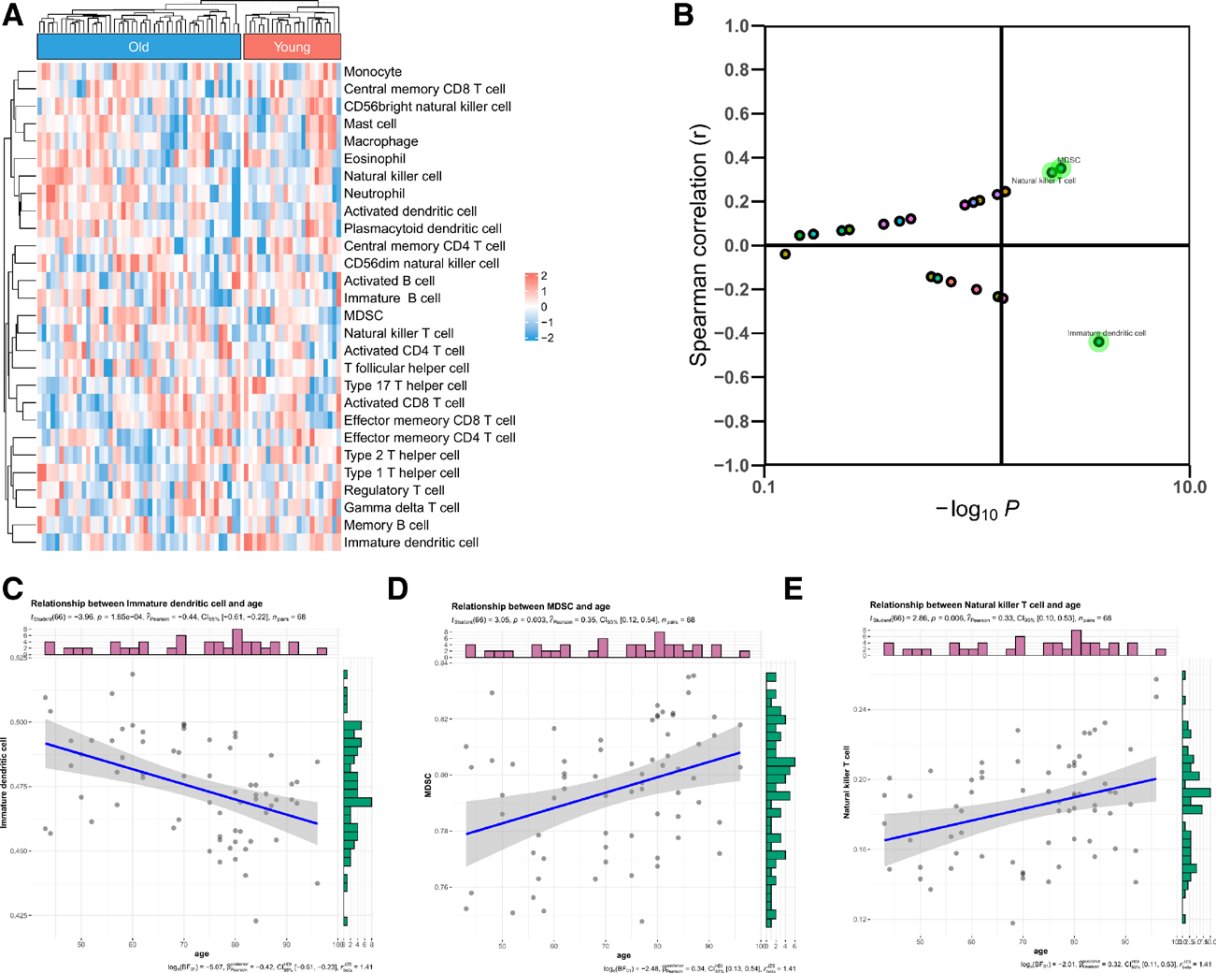
Ageing was associated with immune infiltration. (A, B) Correlation between age and the immune cells. (C-D) The immature dendritic cell is significantly negatively correlated with age while the myeloid-derived suppressor cells (MDSC) and the natural killer T cell are significantly positively correlated with age.

Using Spearman correlation, the relationship between age and immune cell infiltration was analyzed. Correlation analysis revealed that both the Myeloid-derived suppressor cells (MDSC) (*R* = 0.35, *P* = .003) and the natural killer T (NKT) cells (*R* = 0.33, *P* = .006) are significantly positively correlated with age. The immature dendritic cells (R = -0.44, *P* < .001) are significantly negatively correlated with age (Fig. 7).

## 4. Discussion

Cardiovascular disease is a major cause of death in people older than 65 years.^[8]^ Stroke is the main cause of morbidity and mortality in the United States and other developed countries. Its incidence rate is 8%, and the annual incidence rate in elderly people is 2% to 8.8%.^[9]^ However, the molecular mechanisms and immune microenvironment characteristics of elderly patients with stroke are not clearly understood. GO analysis revealed that the response to the virus, type I interferon signaling pathway, focal adhesion, cell-substrate adherens junction, and cytosolic ribosome pathway were enriched. GSEA analysis enriched heme metabolism, interferon gamma response, and interferon alpha response pathways.

Many studies have reported that aged stroke patients are associated with the type I interferon signaling pathway, which is consistent with the results of our study.^[10]^ Type I interferon is an important antiviral cytokine that induces a variety of interferon-stimulated genes.^[11]^ P. Androvic et al^[10]^ reported that the comparison of transcriptional changes in young and old mice 3 days after stroke was significantly enriched in gene ontology terms of type I interferon signaling. Zhang et al^[12]^ have reported that blocking the IFN-1 pathway improved stroke outcomes in young mice. McDonough et al^[13]^ reported that type I interferon can affect microglial phenotype and white matter pathology after stroke.

Subsequently, the top 10 hub genes (IFI27, human leucocyte antigen-G, IFIT2, OAS2, IFI6, interferon alpha-inducible protein 44-like, IFIT3, IRF5, myxo virus resistant 1, and interferon-induced protein with tetratricopeptide repeats 1) were identified. The gene-miRNA network was constructed, and the top 2 miRNAs targeted genes are IFI27 and OAS2. IFI27 is a protein-coding gene that could promote cell death and induce apoptosis.^[14,15]^ OAS2 is an interferon-induced antiviral enzyme, which plays a key role in the cellular innate antiviral response.^[16–18]^ Numerous studies have shown that both IFI27 and OAS2 are involved in the interferon pathway. Ugolini–Lopes reported that IFI27 is in the type I IFN signature, which was associated with the young age of antiphospholipid syndrome.^[19]^ Zhang et al^[20]^ reported that OAS2 could regulate the IFN-I signaling pathway in nasal swabs and lung tissues infected with severe acute respiratory syndrome coronavirus 2.

This study used ssGSEA and Spearman correlation to reveal the relationship between age and immune infiltration level in stroke patients. We found that age was positively correlated with MDSC and NKT cells and negatively correlated with immature dendritic cells. NKT is a bridge between innate and adaptive immunity.^[21]^ It can express T cell receptors and NK cell lineage receptors.^[21]^ Many studies have shown that temporary or permanent blocking of the middle cerebral artery (tMACO or pMACO) can increase NK cell infiltration in the ischemic hemisphere.^[22,23]^ Liu et al^[24]^ showed that RIPostC significantly inhibited the proportion of NKT cells in the spleen and played a neuroprotective role after stroke in mice. Thus, immune cell infiltration may play an important role in older patients with stroke. Overall, the analysis of age-stroke-related genes showed that the inflammatory environment of elderly animals was enhanced, which was related to the high infiltration and activation of NKT cells and MDSC, and the activation of the type I interferon signaling pathway.

## 5. Conclusions

In this study, 240 differentially expressed genes (DEGs) were identified. GO and GSEA analyses identified that the type I interferon signaling pathway was enriched in older stroke patients. IFI27 and OAS2 have been identified as hub genes and have been reported to be associated with type I interferon signaling pathways in many studies. Immune infiltration analysis showed that aging stroke was associated with NKT cells, which have been reported to be associated with the type I interferon signaling pathway in stroke. In conclusion, our study suggests that aging stroke may be regulated by IFI27 and OAS2 via the type I interferon signaling pathway. Therefore, the specific molecular mechanisms of these genes require further investigation.

## Author contributions

**Conceptualization:** Xu He, Jianping Gu,Jie Kong.

**Data curation:** Yisheng Peng, Zhengli Liu, Guanqi Fu.

**Formal analysis:** HaoBo Su, Wensheng Lou,Guoping Chen,Jie Kong.

**Funding acquisition:** Jianping Gu,Jie Kong.

**Investigation:** Yisheng Peng, Zhengli Liu, Guanqi Fu.

**Methodology:** Xu He, Jianping Gu,Jie Kong.

**Project administration:** Xu He, Jianping Gu,Jie Kong.

**Resources:** Yisheng Peng, Zhengli Liu,Guanqi Fu.

**Software:** Boxiang Zhao, Maofeng Gong.

**Supervision:** Xu He, Jianping Gu.

**Validation:** Xu He, Jianping Gu.

**Visualization:** Zhaoxuan Lu, Yangyi Zhou, Liang Chen.

**Writing – original draft:** Yisheng Peng,Jie Kong.

**Writing – review & editing:** Xu He, Jianping Gu.
